# Peste des Petits Ruminants Virus in Heilongjiang Province, China, 2014

**DOI:** 10.3201/eid2104.141627

**Published:** 2015-04

**Authors:** Jingfei Wang, Miao Wang, Shida Wang, Zaisi Liu, Nan Shen, Wei Si, Gang Sun, Julian A. Drewe, Xuehui Cai

**Affiliations:** Harbin Veterinary Research Institute, Harbin, China (J. Wang, S. Wang, Z. Liu, N. Shen, W. Si, X. Cai);; Heilongjiang Institute of Animal Health Inspection, Harbin, China (M. Wang, G. Sun);; Royal Veterinary College, London, UK (J.A. Drewe)

**Keywords:** peste des petits ruminants, PPR, peste des petits ruminants virus, PPRV, viruses, sheep, goats, ruminants, Heilongjiang Province, China

## Abstract

During March 25–May 5, 2014, we investigated 11 outbreaks of peste des petits ruminants in Heilongjiang Province, China. We found that the most likely source of the outbreaks was animals from livestock markets in Shandong. Peste des petits ruminants viruses belonging to lineages II and IV were detected in sick animals.

Peste des petits ruminants (PPR) is a contagious disease that infects goats and sheep and has a case-mortality rate of ≈80% for acute cases. PPR virus (PPRV) is a member of the family *Paramyxoviridae*, genus *Morbillivirus* (*1*). The disease is present mainly in Africa, the Middle East, and the Indian subcontinent (*2*–*8*).

In July 2007, a PPR outbreak was reported in the Ngari region of southwestern Tibet, China. The outbreak was eliminated by using strict control measures. These measures included culling of all animals suspected to be infected; using a PPR vaccine (75/1 strain) (Tecon Animal Husbandry Bio-Technology Co. Ltd., Xinjiang, China) throughout Tibet and neighboring areas; and restriction of transport of animals (*9*).

On December 5, 2013, a new outbreak of PPR was reported in Huocheng County in Xinjiang Province. PPR was also detected in Gansu, Inner Mongolia, Ningxia, Jiangxi, and Hunan Provinces by mid-March 2014.

Heilongjiang Province is the northernmost province of China (Figure 1, panel A). The total population of small ruminants in this province was ≈9 million in 2012 (http://www.stats.gov.cn), and nearly all ruminants were raised for meat or wool production. Sheep and goats were always kept separately, and registered flocks contained 120–6,000 animals (mean 434 animals). Before 2014, no PPR outbreaks had been documented in this region. We report outbreaks of PPR in 11 counties in Heilongjiang Province during 2014.

**Figure 1:**
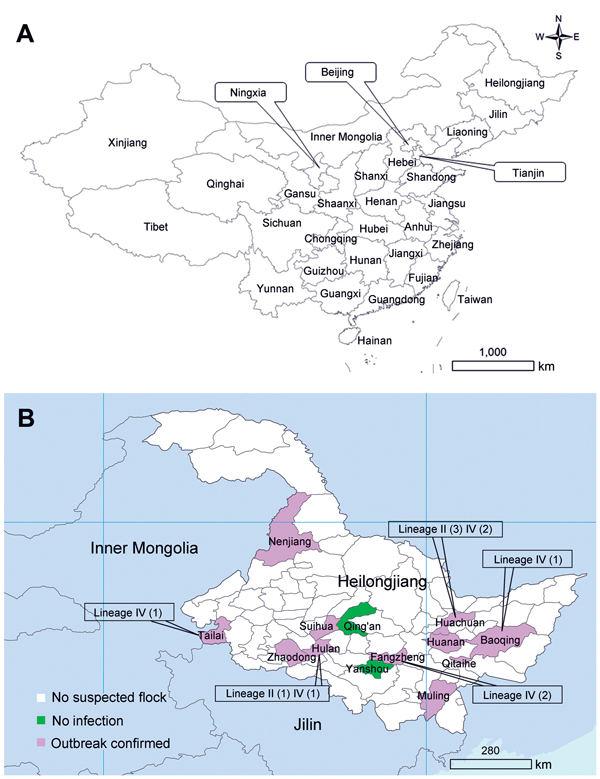
A) Provinces of China and B) distribution of confirmed outbreaks of peste des petits ruminants and peste des petits ruminants virus detected in Heilongjiang Province, March 25–May 5, 2014. Values in parentheses are number of virus isolates.

## The Study

On March 25, 2014, health authorities reported that 43 of 84 goats on 5 breeding farms had died in Huanan County in Heilongjiang Province after being transported from Jiaxiang County in Shandong Province on March 13. Animals showed clinical signs and symptoms of PPRV infection, including fever (temperature >39.5°C), ocular and nasal discharge, pneumonia, and severe diarrhea. On March 26, health authorities reported that 13 of 87 imported breeding goats died of suspected PPR in Huachuan County, which also borders Huanan County. These animals had also been transported from Jiaxiang County on March 13. Because of this animal deaths, a systematic investigation of suspected PPR outbreaks was launched by the Center for Animal Disease Control and Prevention of Heilongjiang Province.

After confirmation of the first case of PPR, active clinical surveillance was conducted by the local veterinary authorities, and farmers were urged to report suspected cases. Suspected flocks were defined as those that 1) during the previous month had ≥1 sheep or goats with ≥1 signs of illness: fever (temperature >39.5°C), depression, anorexia, ocular and nasal discharge, pneumonia, necrosis and ulceration of mucous membranes, and severe diarrhea; 2) had sheep or goats transported from other provinces, especially Shandong Province; or 3) had neighboring farms on which animals had suspected PPRV infections.

During March 25–May 5, a total of 141 suspected flocks (of which 41 contained imported animals [defined as imported flocks]; other flocks were defined as local flocks) from 13 counties were investigated. Sources of all introduced animals were livestock markets in Shandong Province, which were located mainly in 3 counties (Jiaxiang, Heze, and Liangshan). Because spring is traditionally the time when farmers increase numbers of animals on their farms, breeding sheep or goats are bought from livestock markets for this purpose. Shandong Province has the largest number of small ruminant markets in China. It takes 3–5 days for animals to be transported by truck from these markets to most destinations.

From the 141 suspected flocks, 1,887 serum samples were randomly collected from clinically healthy animals, and 285 nasal swab and 28 tissue samples (lymph node, spleen, lung, and intestine) were collected from all ill or dead animals. Competitive ELISAs were performed to detect antibodies against PPRV in the serum samples as described (*10*,*11*).

Viral genomic RNA was extracted from nasal swab and tissue samples by using the QIAamp Viral RNA Mini Kit (QIAGEN, Valencia, CA, USA) according to the manufacturer’s instructions. PPRV was detected by using a reverse transcription PCR (RT-PCR) specific for the 3′ end of the nucleoprotein gene of PPRV; this RT-PCR yields an amplification product of 351 bp (*12*). PCR products were purified by using the QIA Quick Gel Extraction Kit (QIAGEN) and sequenced.

Multiple sequence alignment was performed by using ClustalX2.0 (*13*), and a phylogenetic tree was constructed by using the neighbor-joining method with MEGA6 (*14*). A bootstrap analysis of 1,000 replicates was performed to test the degree of branching.

Serologic analysis indicated that 17% (312/1887) of sampled animals and 76% (31/41) of imported flocks had antibodies against PPRV; all animals from Suihua, Yanshou, and Qing’an Counties were antibody negative (Table). A total of 29% (29/100) of local flocks in 5 counties (Zhaodong, Hulan, Huachuan, Nenjiang, and Baoqing) had antibody-positive animals, which suggests that transmission of PPRV between local and imported flocks had occurred in these areas.

**Table:** Serologic and molecular diagnosis for peste des petits ruminants virus in 13 counties in Heilongjiang Province, China, March 25–May 5, 2014*

	No. flocks/no. tested (%)							No. samples/no. tested (%)				Lineage (no. viruses detected)
County	Antibody positive				PCR positive			Antibody-positive		PCR-positive		
	Imported	Local			Imported	Local		Serum		Nasal swab	Tissue	
Baoqing	10/10 (100)		11/70 (16)		10/10 (100)	8/70 (11)		146/533 (27)		11/146 (8)	10/10 (100)	IV (1)
Hulan	1/2 (50)		8/14 (57)		1/2 (50)	2/14 (11)		38/315 (12)		4/24 (17)	1/2 (50)	II (1), IV (1)
Zhaodong	1/1 (100)		4/5 (80)		1/1 (100)	2/5 (40)		30/222 (14)		6/25 (24)	1/1 (100)	0
Huanan	5/5 (100)		0/3 (0)		1/5 (20)	0/3 (0)		3/157 (2)		0/13 (0)	2/2 (100)	0
Huachuan	6/6 (100)		1/1 (100)		4/6 (67)	0/1 (0)		29/130 (22)		11/20 (55)	2/2 (100)	II (3), IV (2)
Tailai	1/3 (33)		0/2 (0)		1/3 (33)	0/2 (0)		24/111 (22)		0/12 (0)	1/1 (100)	IV (1)
Nenjiang	2/2 (100)		5/5 (100)		2/2 (100)	3/5 (60)		16/99 (16)		5/15 (33)	1/1 (100)	0
Qitaihe	3/3 (100)		0		2/3 (67)	0		14/79 (18)		1/15 (7)	1/1 (100)	0
Fangzheng	1/1 (100)		0		1/1 (100)	0		11/70 (16)		1/12 (8)	2/2 (100)	IV (2)
Suihua	0/1 (0)		0		1/1 (100)	0		0/52 (0)		0/3 (0)	3/3 (100)	0
Muling	1/1 (100)		0		1/1 (100)	0		1/2 (50)		0	1/3 (33)	0
Yanshou	0/3 (0)		0		0	0		0/67 (0)		0	0	0
Qing’an	0/3 (0)		0		0	0		0/50 (0)		0	0	0
Total	31/41 (76)		29/29 (100)		25/35 (71)	15/15 (100)		312/1,887 (16)		39/285 (14)	25/28 (89)	II (4), IV (7)

*An antibody-positive flock contained ≥1 animal in positive for antibody against peste des petits ruminants virus. A PCR-positive flock contained ≥1 animal positive for peste des petits ruminants virus by PCR.

RT-PCRs showed that 14% (39/285) of nasal swab samples and 89% (25/28) of tissue samples were positive for the nucleoprotein gene of PPRV; overall PPRV positive rate was 20% (64/313) (Table). Combined results of serologic and the molecular diagnostic tests indicated that 43% (60/141) of suspected flocks were positive for PPRV. Eleven outbreaks were confirmed in Heilongjiang Province; the geographic distribution of these outbreaks is shown in Figure 1, panel B.

**Figure 2:**
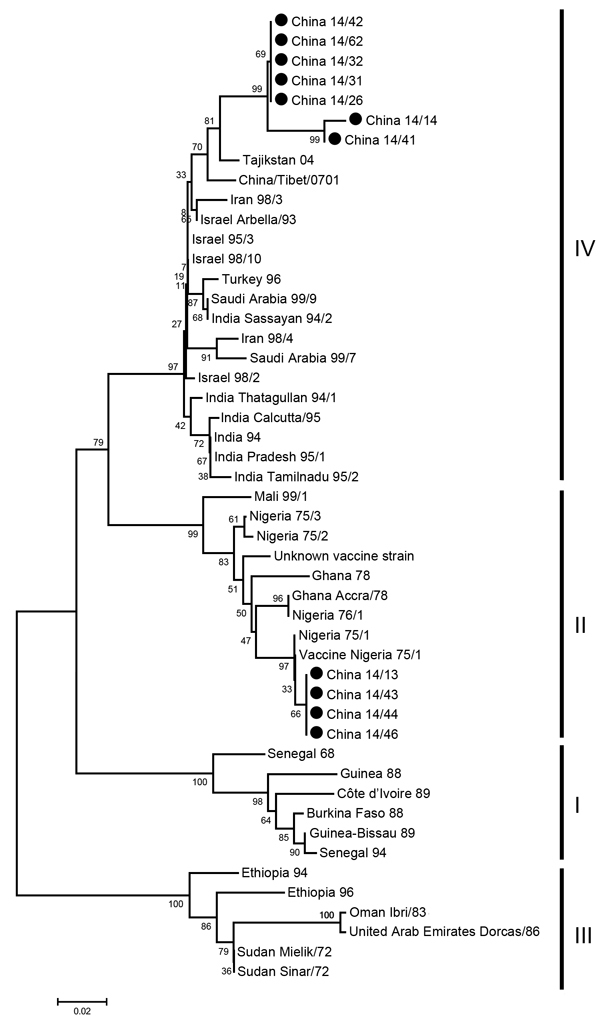
Phylogenetic analysis of sequences of the 3′ ends of nucleoprotein genes of peste des petits ruminant virus (PPRV), Heilongjiang Province, China, March 25–May 5, 2014. The tree was constructed by using the neighbor-joining method in MEGA6 (*14*). Values along branches indicate bootstrap values of 1,000 replicates, and numbers on the right indicate lineages. Black dots indicate PPRV-positive samples isolated in this study. Scale bar indicates estimated number of substitutions per 20 nt.

To identify genetic characteristics of PPRVs in Heilongjiang Province, a phylogenetic tree was generated by using the 3′ ends of nucleoprotein genes of 49 viruses: 11 were obtained from RT-PCR–positive samples in this study and 38 were obtained from GenBank. Seven of the 11 viruses belonged to lineage IV and were closely related to the strains Tajikistan/04 (*12*) and Tibet/07 (*9*). The remaining 4 viruses from this study belonged to lineage II and were closely related to vaccine strain Nigeria/75/1 (Figure 2). All 11 viruses were obtained from sick animals. These 11 animals had not been vaccinated in Shandong or Heilongjiang Provinces; previous vaccination information was not available.

To control and prevent further spread of PPR, several measures were initiated by the animal health authorities in Heilongjiang Province. These measures included 1) culling of all animals in PPRV-infected flocks and on all farms within a radius of 5 km; 2) restriction of transport of sheep and goats throughout the province; 3) disinfection of PPRV-contaminated areas and disposal of dead animal carcasses; and 4) enhancement of the animal disease–reporting mechanisms. Before the PPR outbreak, sheep and goats with signs and symptoms of disease were not required to be reported to the local veterinary authorities, except for animals with foot-and-mouth disease. After the outbreak, farmers were required to report all sick animals and vaccination of all susceptible animals. These control measures eventually contained the outbreaks.

## Conclusions

The emergence of PPR in Heilongjiang Province poses a great threat to the livestock industry in this province. Our investigation showed that the most likely source of the outbreaks was animals from livestock markets in Shandong Province. This finding suggests that transportation of unidentified PPRV-infected sheep and goats is a major risk factor for spread of PPR in China.

Lineage II and lineage IV PPRVs were detected in sick animals. Although the partial nucleoprotein gene of the lineage II viruses had similar sequence identity (>99%) to that of vaccine strain 75/1, we could not determine the vaccination status of these sick animals. Future studies should focus on determining the virulence of these viruses.
